# *Mycobacterium tuberculosis *spoligotypes and drug susceptibility pattern of isolates from tuberculosis patients in peri-urban Kampala, Uganda

**DOI:** 10.1186/1471-2334-8-101

**Published:** 2008-07-28

**Authors:** Benon B Asiimwe, Solomon Ghebremichael, Gunilla Kallenius, Tuija Koivula, Moses L Joloba

**Affiliations:** 1 Department of Medical Microbiology, Makerere University Medical School, P.O Box 7072, Kampala, Republic of Uganda; 2 Department of Microbiology, Tumor and Cell Biology, Karolinska Institutet, SE-171 77, Stockholm, Sweden; 3 Department of Bacteriology, Swedish Institute for Infectious Diseases Control, SE-171 82, Solna, Sweden

## Abstract

**Background:**

The poor peri-urban areas of developing countries with inadequate living conditions and a high prevalence of HIV infection have been implicated in the increase of tuberculosis (TB). Presence of different lineages of *Mycobacterium tuberculosis *has been described in different parts of the world. This study determined the predominant strain lineages that cause TB in Rubaga division, Kampala, Uganda, and the prevalence of resistance to key anti-tuberculosis drugs in this community.

**Methods:**

This was a cross-sectional study of newly diagnosed sputum smear-positive patients aged ≥ 18 years. A total of 344 isolates were genotyped by standard spoligotyping and the strains were compared with those in the international spoligotype database (SpolDB4). HIV testing and anti-tuberculosis drug susceptibility assays for isoniazid and rifampicin were performed and association with the most predominant spoligotypes determined.

**Results:**

A total of 33 clusters were obtained from 57 spoligotype patterns. According to the SpolDB4 database, 241 (70%) of the isolates were of the T2 family, while CAS1-Kili (3.5%), LAM9 (2.6%), CAS1-Delhi (2.6%) were the other significant spoligotypes. Furthermore, a major spoligotype pattern of 17 (4.5%) strains characterized by lack of spacers 15–17 and 19–43 was not identified in SpolDB4. A total of 92 (26.7%) of the patients were HIV sero-positive, 176 (51.2%) sero-negative, while 76 (22.1%) of the patients did not consent to HIV testing. Resistance to isoniazid was found in 8.1% of strains, while all 15 (4.4%) strains resistant to rifampicin were multi-drug resistant. Additionally, there was no association between any strain types in the sample with either drug resistance or HIV sero-status of the patients.

**Conclusion:**

The TB epidemic in Kampala is localized, mainly caused by the T2 family of strains. Strain types were neither associated with drug resistance nor HIV sero-status.

## Background

Uganda is one of the countries with the highest burden of tuberculosis (TB) in Sub-Saharan Africa, with an estimated incidence of 559 cases per 100,000 per year and ranks 16^th ^among the 22 high-burden countries [1]. Kampala, the capital of Uganda, has an approximate population of 2 million (Nation Census, 2002) and accounts for 30% of the TB burden in the country (National Tuberculosis and Leprosy Control Programme, 2006). To date, there are very limited data available pertaining strains circulating in Uganda and the East African region as a whole [2-5]. Poor peri-urban areas of most developing countries where living conditions are unsatisfactory are usually affected by TB [6,7]. The presence of Human immunodeficiency virus (HIV) has caused an increase in *Mycobacterium tuberculosis *complex (MTC) infection [8] and rapid progression of the infection [9], and is also known to increase MTC transmission rates at the community level, further threatening the health and survival of HIV sero-negative individuals as well [10].

Spoligotyping, one of the genotyping techniques, is a simple, rapid and cost-effective method that has been used widely to define predominant and a growing number of important clades worldwide [2,3,11-13]. The World spoligotyping Database, SpolDB4.0, describes an update on the global distribution of *M. tuberculosis *complex spoligotypes but shows little information about Uganda. A previous characterization of 234 MTC strains collected between 1995 and 1997 at the National Referral Hospital in Kampala grouped 67% of the isolates into two closely related spoligotype families [4] but provided neither an estimate of the burden of other strain lineages and associated HIV sero-status data nor the anti-tuberculosis susceptibility pattern of the isolates.

The present study has characterized isolates from a peri-urban patient population in Rubaga division, Kampala. We have assigned 244 of the 344 strains to the major clades in SpolDB4.0, and also investigated association between the predominant spoligotypes and HIV sero-status as well as anti-tuberculosis drug resistance in this community.

## Methods

### Ethical considerations

Institutional permission to conduct the study was obtained from the Faculty Research and Ethics Committee of Makerere University Medical School (institutional ethics board of the Medical School). Informed consent to participate in the study as well as permission to use isolates from samples provided were obtained from all enrolled participants.

### Study setting

Patients were recruited from sputum smear positive residents in Rubaga attending four main TB clinics in the division. Sample processing, confirmatory microscopy and culture and susceptibility testing were performed at the National Tuberculosis and Reference Laboratory (NTRL); molecular assays were run at Makerere Medical School, and data analysis for spoligotyping was done at the Swedish Institute for Infectious Disease Control, Stockholm.

### Study design

This was a cross sectional study in which all consecutive newly-presenting Ziehl-Neelsen (ZN) smear-positive patients between February and November 2006 aged 18 years and above were enrolled. The patient recruitment and sample collection process was done as described in our previous report [14]. Briefly, all patients underwent a standardized interview and three consecutive sputum samples (spot, early morning and spot) were taken from each patient. Only the sample with the highest ZN smear grade was further analyzed for each patient. Samples were stored at 4°C at the recruitment clinics, in any case for not more than 48 hours, until transported in a cold box to the NTRL for processing, confirmatory fluorescent microscopy and culture.

### Sputum sample processing

Specimens (2.5–10 ml) were processed by the standard N-acetyl L-cystein (NALC)-NaOH method [15] and concentrated at 4000 × *g *for 15 minutes. The sediment, irrespective of the original sample volume, was reconstituted to 2.5 ml with phosphate buffer pH 6.8, to make the inoculum for the smears and cultures.

### Culture and identification

Two Lowenstein-Jensen slants, one containing 0.75% glycerol and the other containing 0.6% pyruvate, were inoculated with the sediment and incubated at 37°C. Cultures were considered negative when no colonies were seen after 8 weeks incubation. Isolates were harvested, DNA extracted using a standardized protocol [16] and confirmed as MTC by an in-house PCR [17].

### Drug resistance assays

Drug susceptibility testing (DST) was performed by the indirect proportion method on Lowenstein-Jensen media at the following final drug concentrations: rifampicin, 40 μg/ml and isoniazid, 0.2 μg/ml, as recommended elsewhere [18]. Multi-drug resistance (MDR) was defined in accordance with standard criteria of resistance to at least isoniazid and rifampicin.

### Quality control for DST

For all test panels, drug susceptible strain (H37Rv) and specific drug resistant strains (TMC 303 for isoniazid and TMC 331 for rifampicin) internal controls were included.

The NTRL successfully participates in two annual external proficiency testing programmes organized by Centers for Disease Control and Prevention (CDC) and the Supra national laboratory net work.

### Spoligotyping

Standard spoligotyping [19] was done generally as described by Kamerbeek and colleagues using a commercially available kit (Isogen Bioscience BV, Maarssen, The Netherlands).

### HIV testing

HIV-1 rapid testing was performed according to the Ministry of Health, Uganda, algorithm. Anticoagulated whole blood was immediately used for HIV rapid testing at the clinics of enrolment. Two rapid HIV tests, Unigold Recombinant HIV (Trinity Biotech, Wicklow, Ireland) and Determine HIV-1/2 (Abbott, Tokyo, Japan), were run sequentially. Samples were tested first with Abbot Determine and reported only when negative. Positive samples were confirmed with Unigold, while discordant results were resolved by a third rapid test kit, HIV-1/2 Stat-Pak (ChemBio, Medford, NY). All tests were performed and interpreted according to the manufacturers' instructions.

### Data analysis

Spoligotypes were analysed by a BioNumerics software, version 5.0 (Applied Maths, Kortrijk, Belgium) as character types. The obtained signatures were compared in binary format with the international spoligotyping database of the Pasteur Institute of Guadeloupe. The SpolDB4 database [20] (an online version is available on ) provides information on the spoligotype international type (SIT) distributions of *M. tuberculosis *spoligotypes worldwide. Labels for major phylogenetic clades were assigned according to signatures provided in SpolDB4. Statistical associations between strain types, drug susceptibility data and HIV sero-status were generated by Stata 8.0 using the Pearson's chi-square test. Odds ratios were estimated at 95% confidence intervals, and a *P *value of < 0.05 was considered evidence of a significant difference.

## Results

### Study population and samples analyzed

Between February and November 2006, 2639 TB suspects from Rubaga division, Kampala, were screened at 4 division TB clinics. The population of Rubaga division is mainly low to middle income, and the clinics chosen are the subsidized mission founded hospitals at which a majority of the residents seek medical care. Only 386 of these suspects were sputum smear positive and enrolled. Extra samples from randomly selected patients were taken for internal quality control as generally described in our previous report [14]. The demographic information of the patients shows that 163 (47.4%) of the isolates were from female patients while 181 (52.6%) were male. The sample median age was 32 with a range of 18 to 62 years. The median age among female subjects was 30 (SD 8.3) with a range of 18 to 60 years while that of the males was 34 (SD 8.6) with a range of 18–62 years. Stratification according to age showed that 267 (77.7%) of the patients were between 18 and 39 years old while 75 (22%) were between 40 and 60 years. Only 1 (0.3%) patient was over 60 years of age.

### Spoligotypes

To determine the strain lineages present in the sample, the 344 isolates were spoligotyped and binary outcomes compared with those existing in SpolDB4 so as to assign spoligotype international type (SIT) designations. A total of 320 isolates, or 93% of our sample, were grouped into 33 clusters (2 to 49 isolates per cluster), while the remaining 24 (7%) of the strains did not cluster. Of these 24 strains that did not cluster, 21 did not exist in the SpolDB4.0 data base, hence represented the true orphans in the study sample. The remaining 3 of the unclustered isolates included one *M. bovis *strain and were all present in SpolDB4 with labels SIT 34 (S), SIT 61 (LAM10-CAM), and SIT 482 (BOV1) for the *M. bovis *strain.

**Figure 1:**
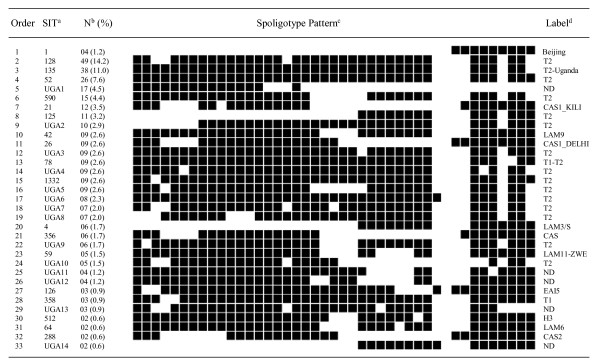
Spoligotype pattern of clustered *M. tuberculosis *strains in the study. ^a^As identified in SpolDB4.0; SIT, spoligotype international type; N^b^, number of isolates (as a percentage of total *M. tuberculosis *strains in the study); ^c^filled boxes represent positive hybridization while empty boxes represent absence of spacers; ^d^label defining the lineage/sub lineage; ND, not yet determined in SpolDB4.0.

Among the 33 clusters, seven included more than ten isolates each and were defined as major spoligotypes, while minor spoligotypes, on the other hand, were defined as spoligotype international types (SITs) that contained two to ten isolates per cluster (Figure 1). Analysis of the frequency of the spoligotypes in our study with SpolDB4.0 allowed differentiation between ubiquitous types (SIT 1, SIT 4, SIT 21, SIT 26, SIT 42, SIT 52, SIT 59, SIT 78, SIT 356, and SIT 288) and those believed to be endemic in Uganda (SIT 125, SIT 128, SIT 135, and SIT 590). Furthermore, up to 14 clusters ranging from two to seventeen isolates per cluster formed a total of 100 strains and were not yet defined in SpolDB4.0. These were labeled UGA1 (17 isolates) to UGA14 (2 isolates) (Figure 1). Seventy percent (241/344) of the isolates lacked hybridization to either spacer 40 or both 40 and 43, and these were characteristic of strains that were previously classified as *M. africanum *genotypes Uganda II and I respectively [21]. Most (227/241) of the Uganda genotype strains formed 16 clusters (ranging 5 to 49 isolates) with 80 strains being genotype I while 147 were genotype II (Figure 1). Only 14 Uganda genotype strains did not cluster.

Overall, 4 Beijing strains (SIT 1) were identified, making 1.2% of the sample. The six major shared spoligotypes in our sample were SIT 128 or T2 with 49 isolates, SIT 135 (T2-Uganda) with 38 isolates, SIT 52 (T2) with 26 isolates, SIT 590 (T2) with 15 isolates, SIT 21 (CAS1-Kili) with 12 isolates and SIT 125 (T2) with 11 isolates. Additionally, one other major cluster of 17 isolates with a characteristic lack of hybridization to spacers 15–17 and 19–43 is not yet defined in SpolDB4.0 and was labeled UGA 1 (figure 1).

### HIV sero-status

In the current sample, 92 patients (26.7%) were HIV sero-positive, 176 (51.2%) sero-negative, while 76 (22.1%) did not consent to HIV testing hence their status unknown. There was no significant difference (P = 0.116) in sero-status between female and male patients in the sample. An analysis of the predominant spoligotypes in HIV sero-positive and sero-negative patients showed that 53/92 (57.6%) of sero-positive individuals carried strains of the T2 family, while only 6.5% carried the CAS strains, 3.3% LAM3/S, and 4.3% carried unique strains. Furthermore, 138/176 (78.4%) of the HIV sero-negative individuals and 50/76 (65.8%) of those who did not consent carried the T2 family of strains. The other patients carried either minor spoligotypes or those not yet defined in SpolDB4.

### Drug susceptibility patterns

Susceptibility testing results for the two key anti-tuberculosis drugs (isoniazid and rifampicin) showed that resistance to isoniazid was 28/344 (8.1%), that to rifampicin was 15/344 (4.4%), and all the rifampicin resistant isolates were also MDR, being defined as resistance to both isoniazid and rifampicin. 13 strains, including 1 Beijing genotype, were monoresistant to isoniazid. Five patients were recruited with recurrent TB, but all the isolates were susceptible. There was no relationship (p = 0.80) between MDR and HIV sero-positivity. A summary of patient demographic characteristics and associated drug susceptibility pattern is shown in Table 1.

**Table 1:** Demographic characteristics and drug susceptibility pattern of isolates in the study

			**Drug susceptibility pattern**			
			
**Demographic ** **characteristics**		**Total**	**Susceptible**	**Resistant to ** **Isoniazid^a^**	**Resistant to ** **Rifampicin^b^**	**MDR**
Total no. of strains		334	316	13	0	15
Sex	Women	163	153	7	0	3
	Men	181	163	6	0	12
History of TB	New cases	339	311	13	0	15
	Retreatment	5	5	0	0	0
HIV status	Positive	92	84	5	0	3
	Negative	176	164	4	0	8
	Unknown	76	68	4	0	4
Age group (years)	18–39	267	249	7	0	11
	40–60	76	66	6	0	4
	>60	1	1	0	0	0

^a^Resistance to isoniazid other than MDR; ^b^resistance to rifampicin other than MDR; MDR = multidrug resistant strains

Regarding cluster analysis in drug resistant isolates, SIT 52 (T2) had four of the 15 MDR isolates; SIT 128 (T2) three MDR isolates, while SIT 135 (T2-Uganda) had two MDR isolates. The other 6 MDR isolates were distributed as follows: one LAM9 (SIT 42), one UGA7 (T2), one UGA18 and three unique (T2) isolates. Although strains of the T2 family accounted for 13 of the fifteen MDR strains in the sample, there was no statistical relationship (p = 0.25) between this strain type and MDR. The relationship between the different spoligotypes and resistance to rifampicin, isoniazid or both is summarized in Table 2.

**Table 2:** Association of drug resistance to isoniazid and rifampicin with spoligotypes

	**No. (%) strains**				
	
	**Spoligotypes**				**All **
		
**Characteristic**	**LAM9**	**T2**	**Beijing**	**Others**	**spoligotypes**
Sensitivity to two key drugs^a^	6 (1.7)	221 (64.2)	3 (0.9)	86 (25)	316 (91.8)
Multidrug resistance	1 (0.3)	13 (3.8)	0 (0)	1 (0.3)	15 (4.4)
Isoniazid resistance^b^	2 (0.6)	7 (2.0)	1 (0.3)	3 (0.9)	13 (3.8)
Rifampicin resistance^b^	0 (0)	0 (0)	0 (0)	0 (0)	0 (0)
Total	9	241	4	90	344

^a^Isoniazid and Rifampicin; ^b^other than MDR

## Discussion

Characterization of prevailing *M. tuberculosis *lineages and clones focusing on different geographical levels such as continents, countries, regions or cities is important for locating the origin, evolution and spreading dynamics of a particular *M. tuberculosis *clone, which is often difficult to be identified by traditional epidemiological investigations alone. Like most of sub-Saharan Africa, Uganda has a high prevalence of TB infection with peri-urban communities of Kampala recording higher rates than those in the rest of the country [22]. In this region where TB is endemic, it is critical to identify predominant strain types in order to study transmission patterns within communities and to understand the epidemiology of the disease in the country as a whole. This report presents the largest amount of molecular epidemiological data on *M. tuberculosis *isolates from Uganda to date. It is also the first systematic community based study conducted to assess strain diversity, associated HIV sero-status and anti-tuberculosis drug resistance of *M. tuberculosis *complex in a peri-urban population of Uganda.

Our findings on the predominance of the T2 family of strains in Kampala compare well with previous data from the study at Mulago hospital, Kampala, in which 67% of the isolates were identified as lacking hybridization to either spacer 40 or both 40 and 43 [4,21]. Else where in East Africa, a previous study in Kenya found only eight (11%) of 73 isolates to be of the T2 family and its variants, while in northern Tanzania four (3%) of 130 strains were T2-Uganda, frequencies much lower than 70% observed in our sample [3]. It is therefore plausible that the TB epidemic in Kampala is local and well established, and that this strain is well adapted to transmit in the local population. Similar trends have been observed in other studies where local genotypes tend to form a greater proportion of the circulating strains. Cases in point are Guinea Bissau where 199 (51%) of 229 isolates belonged to the Guinea Bissau family [23]; Cameroon where 193 (46.7%) of 413 *M. tuberculosis *isolates belonged to the Cameroon family, LAM10-CAM [24]; Harare, Zimbabwe where 68/214 (31.8%) isolates in one study and 116/246 (47.2%) in another were LAM-ZWE variants [25,26] and in Zambia where 74/114 (65%) isolates were also of the LAM-ZWE family [26]. These are in further agreement with recent findings where it was noted that different strains of *M. tuberculosis *have adapted to specific human populations, and that such local strains are more likely to transmit compared to others [27,28].

Other significant spoligotypes in our study were CAS1-Kili (3.5%), LAM9 (2.6%), CAS1-Delhi (2.6%), LAM3/S (1.7%), CAS1 (1.7%), and LAM11-ZWE (1.5%). In comparison to other studies in the region, the CAS, LAM and EAI families were reported at 37%, 22% and 17% respectively of a total of 147 isolates in a study in Dar es Salaam, Tanzania [2]; while in northern Tanzania, the most predominant families were CAS-Kili (30%), LAM11-ZWE (14.6%), EAI (6.2%), Beijing (5.4%), and CAS1-Delhi, T1 and LAM9 at 3.8% [5]. In Kenya, on the other hand, 35.6% of 73 isolates were of the CAS family, while 11% were LAM [3]. These studies show more success of the CAS, LAM and EAI families in the neighboring East African countries, while in Central Uganda, the T2 family of strains predominates.

A comparison of the prevalence of the Beijing family of strains, highly prevalent in many Asian locations [13], shows that in East Africa, Tanzania has reported 14 cases [2,5], Kenya six [3], while in a recent study of mycobacteria causing human cervical lymphadenitis in pastoral communities in the Karamoja region of Uganda, three isolates with the Beijing spoligotype were identified from 34 biopsies [29], but the susceptibility pattern and associated HIV sero-status of the individuals is not known. In this study, to the best of our knowledge, we report the first four *M. tuberculosis *Beijing strains in Uganda with known anti-tuberculosis drug susceptibility pattern to date, with none of the four strains being MDR. Our results further show that the Beijing family at 1.2% is not common. This result differs from that seen in a study in Kenya in which two of the six Beijing strains in the collection were MDR [3]. In Malawi, 44/1,029 (4.3%) of strains in one study were Beijing and susceptible [30]; in the Dar es Salaam study, the susceptibility pattern of the seven Beijing isolates was not determined [2], while more recently in northern Tanzania, 5.4% of 130 isolates were Beijing and all susceptible. Generally, the trend and susceptibility pattern of the Beijing genotype in sub-Saharan Africa has been described to be one of three: epidemic and associated with drug resistance (high level in South Africa); epidemic but drug sensitive (Malawi); and very low level or absent (parts of Africa) [31]. Our data suggest that the Beijing strains in Kampala are of the low level and susceptibility pattern.

The emergence of drug resistance in the treatment of TB has complicated its management. In the developing countries, lack of resources hinder regular drug resistance surveys, hence the magnitude of this problem remains largely unknown. The last national anti-tuberculosis drug resistance survey in Uganda (1996–1997) on 586 patients [32] indicated that primary resistance to isoniazid was 6.7%, that to rifampicin 0.8%, while MDR was 0.5%. In neighboring Rwanda, the most recent survey results [33] show that of 616 strains from new cases, 6.2% were resistant to isoniazid, 3.9% to rifampicin and 3.9% were multidrug-resistant TB. In northern Tanzania, a study of 111 isolates showed that 9.9% were resistant to isoniazid, 2.7% to rifampicin, with 2.7% being MDR [5]. Our result for resistance to isoniazid at 8.1% is comparable to that in northern Tanzania, while that to rifampicin (4.4%) is comparable to the Rwanda findings.

Regarding cluster analysis, although the T2 family of strains accounted for 13/15 MDR strains, there was no statistical evidence (p = 0.25) to suggest that it might be the driving force of anti-tuberculosis drug resistance in this community. Additionally, as observed in northern Tanzania [5], our results show that HIV sero-status could not be identified with a particular spoligopattern. Furthermore, import of strains from Asia (CAS1-Kili, 3.5%; CAS1-Delhi, 2.6%; CAS1, 1.7%; and CAS2, 0.6%) has not had a major impact on the *M. tuberculosis *population in Kampala. However, together with the single *M. bovis *seen in our sample, there is clearly a diverse set of *Mycobacterium tuberculosis *complex strains circulating in Kampala, although T2 strains are predominant.

## Conclusion

This study provides an insight into the *M*. *tuberculosis *strains circulating in Kampala. We have shown that the T2 family is likely to be responsible for the TB epidemic in this region, and that import of new strains has not had a significant bearing on the burden of disease in Rubaga division. Additionally, strain types in our sample were neither associated with drug resistance nor HIV sero-status.

## Competing interests

The authors declare that they have no competing interests.

## Authors' contributions

BBA participated in the design and conduct of the study, acquisition of samples and demographic data, culture and isolation of mycobacteria, molecular assays, data analysis and drafting of manuscript; SG participated in data analysis and critical revision of manuscript; GK participated in the conception and design of the study, general supervision of the research in Sweden, and critical revision of the manuscript; TK participated in general supervision of the research in Sweden and critical revision of the manuscript; MLJ participated in the conception and design of the study, general supervision of the research in Uganda, and critical revision of the manuscript. All authors read and approved the final version of the manuscript.

## Pre-publication history

The pre-publication history for this paper can be accessed here:


